# 595. Letermovir (LTV) for Secondary Cytomegalovirus (CMV) Prevention in High Risk Hematopoietic Cell Transplant (HCT) Recipients: Interim Results of a Single Center, Open Label Study

**DOI:** 10.1093/ofid/ofab466.793

**Published:** 2021-12-04

**Authors:** Gyuri Han, Anat Stern, Yiqi Su, Boglarka Gyurkocza, Craig Sauter, Brian Shaffer, Genovefa Papanicolaou, Genovefa Papanicolaou

**Affiliations:** 1 Memorial Sloan Kettering Cancer Center, New York, New York; 2 Memorial Sloan Kettering, New York, NY

## Abstract

**Background:**

Letermovir (LTV) is effective for prevention (ppx) of primary clinically significant CMV infection (csCMVi) in the first 100 days after hematopoietic cell transplant (HCT). Data on LTV for secondary ppx is limited. We report on the efficacy and safety of LTV administered for 14 weeks as secondary CMV ppx.

**Methods:**

Patients (pts) enrolled in an open label study of LTV (ClinicalTrials.gov identifier: NCT04017962) from August 2019 through February 2021 were analyzed. Key eligibility criteria were: CMV high risk (receipt of mismatched and/or T-cell depleted HCT and/or graft versus host disease (GVHD) requiring systemic immunosuppressants) AND prior csCMVi with either undetectable CMV (≤ 136 IU/mL) or ≥ 2 consecutive values < 300 IU/mL at enrollment. Pts with breakthrough csCMVi on LTV or history of LTV resistance were excluded. LTV was administered for 14 weeks or csCMVi whichever occurred first. The study duration was 24 weeks. CMV was monitored per standards of care. The primary endpoint was csCMVi by week 14. Secondary endpoints were csCMVi by week 24, LTV resistance, CMV end-organ disease (EOD) and adverse events (AE) at least possibly related to LTV.

**Results:**

Of 20 pts analyzed, the median age was 58 years (interquartile range [IQR] 46-63); 17 (85%) pts were CMV seropositive, 7 (35%) received mismatched HCT (haploidentical 3, cord blood 3; mismatched unrelated 1), 9 (45%) received CD34 selected allograft and 9 (45%) had GVHD at enrollment. Fourteen (70%) pts had received prior LTV. The median time from HCT to enrollment was 156 (IQR 37-244) and 55 (IQR 40-69) days for pts with and without prior LTV, respectively (P=0.16). CMV at enrollment was < 136IU/mL for 8 (40%) pts. By week 14, 4 (20%) pts developed csCMVi at median 48 days (range 40-66). Resistance testing performed in 3 of the 4 pts, identified LTV resistance mutations in 2 pts. There were no AEs related to LTV, and none developed EOD. Two pts developed csCMVi in the follow up phase. Three pts died during follow up (due to relapse, treatment related toxicity and GVHD), and four pts are in follow up.

**Conclusion:**

LTV secondary prophylaxis was safe and prevented recurrent csCMVi in 80% of high risk patients, including patients with prior LTV exposure. Our data supports the utility of LTV for secondary CMV prevention following HCT.

**Disclosures:**

**Boglarka Gyurkocza, MD**, **Actinium Pharma, Inc.** (Grant/Research Support, Research Grant or Support) **Genovefa Papanicolaou, MD**, **ADMA biologics and Siemens Healthineers** (Consultant, Other Financial or Material Support)**AlloVir** (Consultant, Other Financial or Material Support)**Amplyx** (Scientific Research Study Investigator, Other Financial or Material Support)**Astellas** (Consultant, Grant/Research Support, Scientific Research Study Investigator, Research Grant or Support, Other Financial or Material Support)**Behring** (Consultant, Other Financial or Material Support)**Chimerix** (Consultant, Grant/Research Support, Scientific Research Study Investigator, Research Grant or Support, Other Financial or Material Support)**Cidara** (Consultant, Other Financial or Material Support)**Merck & Co** (Consultant, Grant/Research Support, Scientific Research Study Investigator, Research Grant or Support, Other Financial or Material Support)**Partners Therapeutics** (Consultant, Other Financial or Material Support)**Shionogi** (Consultant, Other Financial or Material Support)**Shire/Takeda** (Consultant, Grant/Research Support, Scientific Research Study Investigator, Research Grant or Support, Other Financial or Material Support) **Genovefa Papanicolaou, MD**, allovir (Individual(s) Involved: Self): Consultant; amplyx (Individual(s) Involved: Self): Consultant; behring (Individual(s) Involved: Self): Consultant; Merck&Co (Individual(s) Involved: Self): Consultant, Investigator and received funding and consulting fees from Merck, Chimerix, Shire and Astellas, Research Grant or Support; octapharma (Individual(s) Involved: Self): Consultant; Partners Therapeutics (Individual(s) Involved: Self): Consultant; takeda (Individual(s) Involved: Self): Consultant, Scientific Research Study Investigator

